# Larva of the greater wax moth, *Galleria mellonella*, is a suitable alternative host for studying virulence of fish pathogenic *Vibrio anguillarum*

**DOI:** 10.1186/s12866-015-0466-9

**Published:** 2015-06-23

**Authors:** Stuart McMillan, David Verner-Jeffreys, Jason Weeks, Brian Austin, Andrew P. Desbois

**Affiliations:** Marine Biotechnology Research Group, Institute of Aquaculture, School of Natural Sciences, University of Stirling, Stirling, UK; Centre for Environment, Fisheries and Aquaculture Science, Weymouth, UK; Present address: Department of Environmental Science and Technology, Cranfield University, Bedford, UK

**Keywords:** Alternative host, Atlantic salmon, Replacement, reduction and refinement (3Rs), Vibriosis, Wax moth larvae

## Abstract

**Background:**

Microbial diseases cause considerable economic losses in aquaculture and new infection control measures often rely on a better understanding of pathogenicity. However, disease studies performed in fish hosts often require specialist infrastructure (e.g., aquaria), adherence to strict legislation and do not permit high-throughput approaches; these reasons justify the development of alternative hosts. This study aimed to validate the use of larvae of the greater wax moth (*Galleria mellonella*) to investigate virulence of the important fish pathogen, *Vibrio anguillarum*.

**Results:**

Using 11 wild-type isolates of *V. anguillarum*, these bacteria killed larvae in a dose-dependent manner and replicated inside the haemolymph, but infected larvae were rescued by antibiotic therapy. Crucially, virulence correlated significantly and positively in larva and Atlantic salmon (*Salmo salar*) infection models. Challenge studies with mutants knocked out for single virulence determinants confirmed conserved roles in larva and fish infections in some cases (pJM1 plasmid, *rtxA*), but not all (*empA*, *flaA*, *flaE*).

**Conclusions:**

The *G. mellonella* model is simple, more ethically acceptable than experiments on vertebrates and, crucially, does not necessitate liquid systems, which reduces infrastructure requirements and biohazard risks associated with contaminated water. The *G. mellonella* model may aid our understanding of microbial pathogens in aquaculture and lead to the timely introduction of new effective remedies for infectious diseases, while adhering to the principles of replacement, reduction and refinement (3Rs) and considerably reducing the number of vertebrates used in such studies.

**Electronic supplementary material:**

The online version of this article (doi:10.1186/s12866-015-0466-9) contains supplementary material, which is available to authorized users.

## Background

Aquaculture plays an increasingly important role in global food production, but microbial diseases continue to cause considerable economic losses to producers and impact negatively on animal welfare [1]. To address microbial disease problems, there is a requirement for deeper understanding of the processes involved during infection, as this will lead to new and improved treatments, vaccinations and management practices. To this end, many studies are performed in vertebrate aquatic hosts such as zebrafish (*Danio rerio*); however, these experiments require specialist infrastructure such as aquaria and biosecurity measures to ensure the safety of workers and aquatic animal stocks, particularly for zoonotic pathogens [2]. In addition, whole-animal studies often are costly, do not permit high-throughput approaches, and are covered by strict legislation [3, 4]. Furthermore, good practice prescribes that researchers should adhere to the principles of the 3Rs, i.e., the replacement, reduction and refinement of experiments on animals [5]. Thus, there is strong justification to pursue alternative infection models when studying microbial pathogens of farmed fish. Alternative infection models can be used because many innate immune responses are functionally conserved across phyla, and pathogens often employ similar mechanisms to exploit different hosts [4]. Still, relatively few studies have described alternative infection models for investigating microbial pathogens of fish, but those available include cell culture [6–8], the amoeba *Dictyostelium discoideum* [2], the nematode *Caenorhabditis elegans* [9], the freshwater ciliate *Tetrahymena thermophila* [10], the crustacean *Artemia franciscana* [11] and *D. rerio* larvae [12, 13]. Even so, each model is associated with shortcomings such as the need for time-consuming training to achieve competence, a requirement for tissue culture or aquarium facilities, the lack of important aspects of immune complexity, or being unsuited to the study of certain pathogen virulence traits.

The larva of the greater wax moth (*Galleria mellonella*) is one alternative model attracting much attention in part due to the simplicity and reliability of establishing infections in this insect [14]. Moreover, there is functional similarity in the innate immune responses to invasive infection between insects and fish with respect to pathogen recognition, expression of antimicrobial peptides, generation of reactive oxygen species, phagocytosis of invading microbes, and initiation of clotting cascades [15–19]. The larvae are a convenient size for manipulation (2 to 3 cm in length), do not need feeding, require little space or specialist infrastructure, and are inexpensive to purchase [14]. Furthermore, the techniques needed to work with this model are achieved after only limited instruction, and studies on this model are ethically more acceptable than working with vertebrates [14]. Notably, the *G. mellonella* model presents a low biohazard risk because no liquid systems are required and the larvae are kept in Petri dishes with infected material made safe by autoclaving. In addition, this model has been used successfully to study virulence of various human pathogens and the efficacy of antibiotic therapies [20]. Still, newly proposed alternative models need to be validated for each pathogen to ensure that an infection occurs, virulence correlates with that observed in the native host, and that conserved virulence mechanisms are involved during infection.

Therefore, the aim of this present study was to assess the suitability of *G. mellonella* as an alternative host to investigate the virulence and pathogenicity of fish pathogens. *Vibrio anguillarum* was selected as the pathogen of interest to validate this alternative infection model because a range of strains and genetic resources are available and much is known of its key virulence mechanisms [21–23]. Moreover, this well-studied Gram-negative bacterium infects many farmed species and continues to cause considerable economic losses worldwide [21, 22, 24].

## Methods

### Reagents and culture media

All chemicals and reagents were purchased from Sigma-Aldrich Ltd (Poole, UK) unless stated. All solutions were made with distilled water except that MilliQ (Millipore Ltd, Watford, UK) was used for molecular biology. Phosphate-buffered saline (PBS) was made up according to Desbois and Coote [20]. Bacteria were cultured routinely on 1.5 % (w/v) NaCl-supplemented tryptone soya agar (TSA; Oxoid, Basingstoke, UK) and broth (TSB; Oxoid) or 1 % (w/v) NaCl-supplemented LB agar and broth (Fisher Scientific, Fair Lawn, NJ, USA), whereas Mueller-Hinton broth (MHB; Oxoid) supplemented with 2 % (w/v) NaCl was used for minimum inhibitory concentration (MIC) determinations. Where required, medium was supplemented with 5 μg/mL chloramphenicol (CHL), 80 μg/mL kanamycin (KAN), 2 μg/mL penicillin G (PEN) or 200 μg/mL streptomycin (STR). Water, PBS and culture media were sterilised by autoclaving at 121 °C for 15 min.

### Bacteria

*V. anguillarum* strains (Table 1) were kept routinely at −70 °C in 15 % (v/v) glycerol. All the strains belong to serotype O1, except for *V. anguillarum* M93Sm (and derivatives) which is serotype O2. Before use, bacteria were recovered initially onto appropriate agar, incubated at 22 °C for 48 h, and then single colonies inoculated into broth (Table 1). Cultures were incubated (22 °C; 150 rpm; approximately 12 h) until mid- to late-exponential phase and then bacterial cells were harvested by centrifugation (2700 × g; 15 min; 4 °C). The cell pellet was washed by resuspension in PBS, centrifuged as before, resuspended again in PBS, and then cell density determined by measuring absorbance at 600 nm (A_600_). Bacterial suspensions were diluted with PBS to the desired CFU/mL, and all inocula were serially diluted in PBS in quadruplicate and plated on TSA or LB agar. Vib1 (= 6018/1 = ATCC 43305) is available in reference culture collections and additional experimental data is provided for this strain as a means to facilitate inter-laboratory comparisons.

**Table 1:** *Vibrio anguillarum* isolates and strains used in this study

Isolate/strain^a^	Genotype	Culture medium^b^	Reference
DM16	NB10 derivative carrying an in-frame 3’-end deletion in *flaA*	TSB/TSA + 1.5%NaCl	[27]
JR1	STR^r^, CHL^r^ M93Sm derivative carrying an inactivating insertion in *vah1*	LB agar/broth + 1%NaCl	[28]
KD12	NB10 derivative carrying an in-frame 5’-end deletion in *flaD*	TSB/TSA + 1.5%NaCl	[29]
KD27	NB10 derivative carrying an in-frame 5’-end deletion in *flaE*	TSB/TSA + 1.5%NaCl	[29]
M93Sm	Plasmid deficient; spontaneous STR^r^ mutant of wild type M93 isolated from *Plecoglossus altivelis*	LB agar/broth + 1%NaCl	[41]
NB10	Wild type	TSB/TSA + 1.5%NaCl	[42]
NB10 cured	NB10 cured of pJM1 virulence plasmid	TSB/TSA + 1.5%NaCl	[32]
NB12	CHL^r^ NB10 derivative carrying a inactivating insertion in *empA*	TSB/TSA + 1.5%NaCl	[30]
S123	STR^r^, CHL^r^ M93Sm derivative carrying a inactivating insertion in *rtxA*	LB agar/broth + 1%NaCl	[7]
S183	STR^r^, CHL^r^, KAN^r^ M93Sm derivative double mutant carrying an inactivating insertions in *rtxA* and in-frame deletion in *vah1*	LB agar/broth + 1%NaCl	[7]
Vib1 (= 6018/1 = ATCC 43305)	Wild type	TSB/TSA + 1.5%NaCl	[21]
Vib39 (= 178/90)	Wild type	TSB/TSA + 1.5%NaCl	[21]
Vib44 (= 261/91)	Wild type	TSB/TSA + 1.5%NaCl	[21]
Vib56 (= 601/91)	Wild type	TSB/TSA + 1.5%NaCl	[21]
Vib64 (= A023)	Wild type	TSB/TSA + 1.5%NaCl	[21]
Vib79 (= LMG 12101)	Wild type	TSB/TSA + 1.5%NaCl	[21]
Vib85 (= 87-9-117)	Wild type	TSB/TSA + 1.5%NaCl	[21]
Vib87 (= T265)	Wild type	TSB/TSA + 1.5%NaCl	[21]
Vib88 (= 51/82/2)	Wild type	TSB/TSA + 1.5%NaCl	[21]
Vib93 (= 850610-1/6a)	Wild type	TSB/TSA + 1.5%NaCl	[21]
Vib134 (= 91-8-178)	Wild type	TSB/TSA + 1.5%NaCl	[21]

TSA, tryptone soya agar; TSB, tryptone soya broth; CHL^r^, chloramphicol-resistant; KAN^r^, kanamycin-resistant; STR^r^, streptomycin-resistant

^a^Strain numbers in brackets refer to the nomenclature used by Pedersen et al. [25]

^b^CHL, KAN and STR were added to the medium at the concentrations described in the Materials and Methods for reviving cryopreserved bacteria and preparing inocula for injection

### Insects

*G. mellonella* larvae in their final instar stage were purchased (approximately 220 mg each; UK Waxworms Ltd, Sheffield, UK), stored in the dark at 4 °C, and used within 14 days. Unless otherwise stated, all experiments used groups containing 10 larvae, and most experiments were repeated using larvae from different batches to give *n* = 20. A 50-μL Hamilton syringe (Sigma-Aldrich Ltd) was used to inject larvae into the last left proleg with 10 μL of bacterial suspension, antibiotic solution, or PBS. The syringe was cleaned between experiments with consecutive washes of 1 % (w/v) sodium hypochlorite, 70 % ethanol and sterile water. Two negative control groups were always prepared: one group that underwent no manipulation to control for background larval mortality (no manipulation control) and one group (uninfected control) that was injected with PBS only to control for the impact of physical trauma. There was never more than one death per control group per experiment. Larvae were stored in Petri dishes in the dark at 15 °C for up to 120 h and inspected every 24 h so that percentage survival could be calculated for each group; larvae were considered dead if they did not move after being touched with a sterile inoculation loop.

### Virulence of *V. anguillarum* in *G. mellonella*

The relative virulence of 11 wild-type *V. anguillarum* strains that had been assessed for virulence in an earlier *Salmo salar* infection trial [25] was assessed in the insect model by inoculating with 10 μL of suspensions containing 1 × 10^3^, 1 × 10^5^ and 1 × 10^7^ total CFU. Relative virulence in the insect was calculated as the cumulative area under the Kaplan-Meier plots of the 1 × 10^3^, 1 × 10^5^ and 1 × 10^7^ CFU groups, and this approach was sufficient to discriminate the virulence of each isolate. Then, relative virulence in the insect model was correlated against the 50 % lethal dose values (LD_50_) determined in the *S. salar* model by Pedersen et al. [25]. The virulence of the wild-type *V. anguillarum* strains was assessed in *G. mellonella* at 15 °C because the earlier *S. salar* trials were performed at this temperature, thus mitigating the effects of differential expression of temperature-regulated bacterial virulence factors. To examine the effect of culture filtrates on larval survival, culture supernatant was passed through a sterile polyethersulfone 0.22 μm filter (Millipore, Watford, Herts, UK) and then injected into the larvae as above. Meanwhile, to examine the effects of heat-killed bacteria on larval survival, washed bacterial suspensions were adjusted to 5 x10^9^ CFU/mL with PBS and heat-killed (60 °C; 25 min). Heat killing was confirmed by the absence of colonies forming when 100 μL of bacterial suspension was plated across TSA plates and incubation at 22 °C for 48 h. Heat-killed bacterial suspension (10 μL) was injected into the larvae as above. To confirm that antibiotic therapy could rescue larvae from *V. anguillarum* infection, larvae were inoculated with 1 x10^5^ CFU and then treated at 2 h, 24 h and 48 h with tetracycline (TET: 1 μg/g of larva) in 10 μL PBS according to Desbois and Coote [20]. An additional control group assessed for the toxicity of the TET treatment. The 11 strains of *V. anguillarum* had MICs against TET of 0.0125–0.025 μg/mL, which were determined according to Clinical and Laboratory Standards Institute Approved Standard M07-A8 (2008), except that the assays were performed at 22 °C in NaCl-supplemented MHB (Table 1).

### *V. anguillarum* burden in *G. mellonella* tissues

To assess bacterial burden in larvae infected with the 11 wild-type *V. anguillarum* strains, larvae were injected with 1 x10^5^ CFU. At 2 h, 4 h, 8 h, 24 h, 48 h, 72 h, 96, h and 120 h, four surviving larvae in each group were selected at random for bacterial load determination. The last abdominal segment of each larva was removed with sterile scissors and the haemolymph (approximately 5–20 μL) was harvested. Of this, 5 μL was serially diluted in PBS and plated on TSA + PEN. Importantly, a preliminary investigation of *V. anguillarum* Vib79 had determined that approximately 95 % of bacteria were found in the haemolymph rather than body tissues. In addition to sampling surviving larvae, dead larvae inoculated with Vib1, Vib44, Vib56 and Vib85 were bled and plated at 48 h, while larvae inoculated with Vib93, Vib88 and Vib64 were sampled at 96 h, 120 h and 120 h, respectively (all these larvae were alive at the time point 24 h previous). Groups of unmanipulated larvae and larvae inoculated with PBS only were included as controls. PEN in the agar had no effect on the recovery of *V. anguillarum* but prevented the growth of most contaminating microbes introduced from the larva surface or gut, and any that did form colonies were obvious and did not affect CFU determinations. Even so, a selection of recovered colonies from larvae infected with each strain were confirmed as *V. anguillarum* by: i) plating on thiosulfate-citrate-bile salts-sucrose (TCBS) agar as the formation of yellow colonies are characteristic of *Vibrio* spp. (22 °C; 72 h); ii) positive reaction to the MONO-AQUA agglutination test (Bionor, Skien, Norway), which is specific for *V. anguillarum*; iii) positive amplification by polymerase chain reaction (PCR) of *rpoN* that encodes sigma factor σ54 using forward primer rpoN-ang5’ (5’-gttcatagcatcaatgaggag -3’; [26]) and reverse primer rpoN2SMR (5’-tgccgagcagatcaatatgt-3’). For PCR, single colonies were cultured and the cells collected as above. The cell pellet was suspended in 1 mL of sodium chloride-Tris-EDTA buffer and centrifuged (13,000 × g; 60 s; room temperature). The supernatant was removed and the pellet suspended in 100 μL Tris-EDTA buffer, heated (95 °C for 10 min), and centrifuged (13,000 × g; 60 s; room temperature). The supernatant was collected and DNA quantity and purity was assessed on a NanoDrop spectrophotometer (Thermo Scientific, Wilmington, DE, USA), before freezing at −18 °C until needed. Each 10-μL PCR reaction contained 5 μL of 2X MyTaq mix (Bioline, London, UK), 0.4 μL of each primer at 10 mM, 1 μL of DNA sample at 50 ng/μL, and 3.2 μL water. PCR reactions were run on a Biometra T Professional thermocycler (Goettingen, Germany) at: 95 °C for 1 min; then 30 cycles of 95 °C for 15 s, 62 °C for 15 s and 72 °C for 20 s; and 72 °C for 2 min. A no template control and a reaction containing DNA extracted from *Vibrio ordalii* Vib307 were performed as negative controls. PCR products were run on a 1 % agarose gel containing 0.1 μg/mL ethidium bromide in 0.5 % Tris-acetate-EDTA buffer at 80 V. Each well contained 2 μL of sample, 1 μL 6X loading dye (Thermo Scientific, Loughborough, UK) and 3 μL of water.

### Virulence of *V. anguillarum* knockout mutants

To assess the importance of various virulence factors known to play a role in infection of native hosts, the virulence of isogenic mutant and parent strains were assessed by injecting groups of larvae with 1 x10^5^ CFU and comparing survival in each group. Mutant inocula were cultured in medium containing appropriate concentrations of antibiotics (see above and Table 2). Each pair of parent and knockout mutant had been tested for virulence differences in fish models previously [7, 27–30]. All virulence gene knockouts were located on the bacterial chromosomes. Bacteria were recovered from haemolymph and confirmed as *V. anguillarum* as above. Then the mutant genotypes of a selection of recovered colonies were confirmed by various means as follows. DNA was extracted from *in vitro* cultures and the colonies recovered from the larvae. Specific fragments in these samples were amplified by PCR and run on 1 % agarose gels as above, except that annealing temperatures were altered according to primers used (Additional file 1: Table S1). To test for presence of the pJM1 virulence plasmid, two specific primer pairs were designed against two genes known to be located only on plasmid, *angR* and *fatE*. The detection of PCR products of the two expected sizes confirmed the presence of the plasmid (Additional file 1: Table S1). For mutants created by plasmid insertion into the chromosome, primer pairs were designed against: i) a region of the plasmid ~80–170 bp up- or downstream from the insertion site; and ii) a region of the disrupted gene ~25–105 bp up- or downstream from the insertion site. The detection of a PCR product of the expected size confirmed the presence of the mutation (Additional file 1: Table S1). For mutants created by allelic exchange and in-frame deletion, primer pairs were designed against regions up- and downstream of the affected sites. Thus, parent and mutant strains would be expected to generate fragments of different lengths: mutant KD27 yielded a 589-bp product compared to a 779-bp product from the NB10 parent; DM16 yielded a 735-bp product compared to a 888-bp product from the NB10 parent; KD12 yielded a 419-bp product compared to a 599-bp product from the NB10 parent; and S183 yielded a 2534-bp product compared to a 2789-bp product from the M93Sm parent. Negative control strains were used in each assay to control for non-specific reactions.

**Table 2:** Comparison of virulence of *Vibrio anguillarum* parent and isogenic mutant strains lacking virulence factors in *Galleria mellonella* larva and fish models of infection

Knockout mutant	Parent	Inactivated/missing gene(s)	Gene function	Virulence change of mutant in fish	Virulence change of mutant in larvae	Reference
NB10 cured	NB10	Virulence plasmid (pJM1)	Iron-scavenging function	Not done	↓	[32]
NB12	NB10	*empA*	Metalloprotease	↓ (*Oncorhynchus mykiss*)	↑	[30]
DM16	NB10	*flaA*	Flagellin protein	↓ (*O. mykiss*)	↑	[27]
KD12	NB10	*flaD*	Flagellin protein	↓ (*O. mykiss*)	n.s.	[29]
KD27	NB10	*flaE*	Flagellin protein	↓ (*O. mykiss*)	↑	[29]
S123	M93Sm	*rtxA*	Repeat-in-toxin secreted toxin	↓ (*Salmo salar*)	↓	[7]
JR1	M93Sm	*vah1*	Secreted haemolysin	↓ (*S. salar*)	n.s.	[28]
S183	M93Sm	*rtxA* and *vah1*	Secreted toxin and haemolysin	↓ (*S. salar*)	↓	[7]

↑, virulence of mutant greater than parent; ↓ virulence of mutant less than parent. In the larva model, the virulence change of the mutant compared to the parent was either not significant (n.s.) or significant (*p* < 0.05; Holm’s corrected). *n* = 20

### Statistical analyses

Statistical tests were performed using SPSS v17.0 for Windows (SPSS Inc., Chicago, IL, USA). Larval survival was plotted according to the Kaplan-Meier method. Where desired, survival differences between groups of larvae were compared for significance with the logrank test. *p* < 0.05 was considered to indicate a significant difference between groups and multiple comparisons were accounted for by applying Holm’s correction [31]. Pearson’s correlation coefficient was determined for virulence of the 11 wild-type *V. anguillarum* isolates in *S. salar* and *G. mellonella* models of infection.

## Results

### *V. anguillarum* establishes an infection in *G. mellonella*

**Fig. 1:**
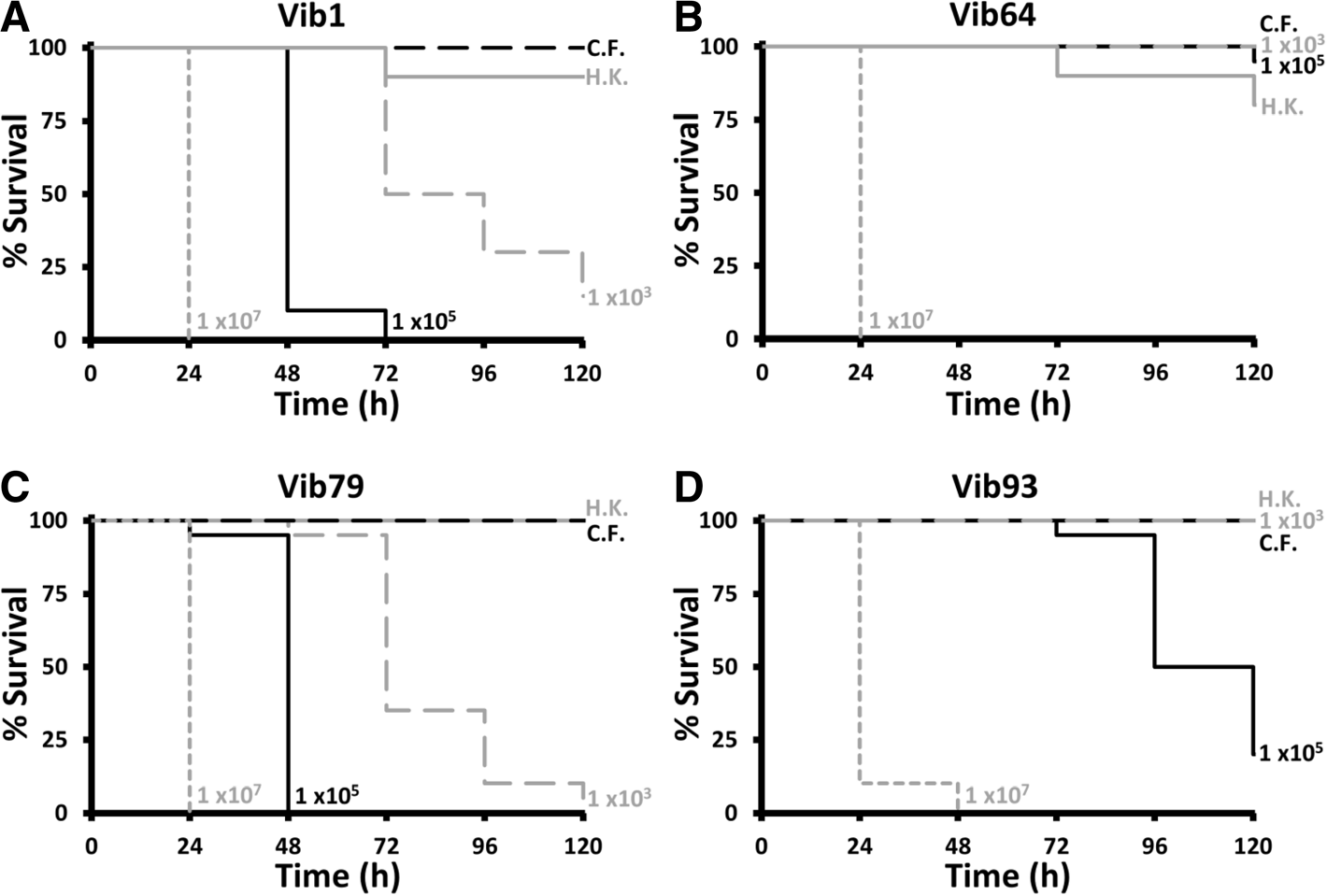
Survival of groups of *Galleria mellonella* larvae injected with culture filtrate (C.F.); 1 × 10^3^, 1 × 10^5^ and 1 × 10^7^ total viable CFU; and 5 × 10^7^ heat-killed (H.K.) CFU of four representative wild-type *Vibrio anguillarum* isolates with different virulence during 120 h, namely Vib1 (**a**), Vib64 (**b**), Vib79 (**c**) and Vib93 (**d**). For clarity, the unmanipulated and uninfected control groups data are not shown. *n* = 20, except H.K. group where *n* = 10

**Fig. 2:**
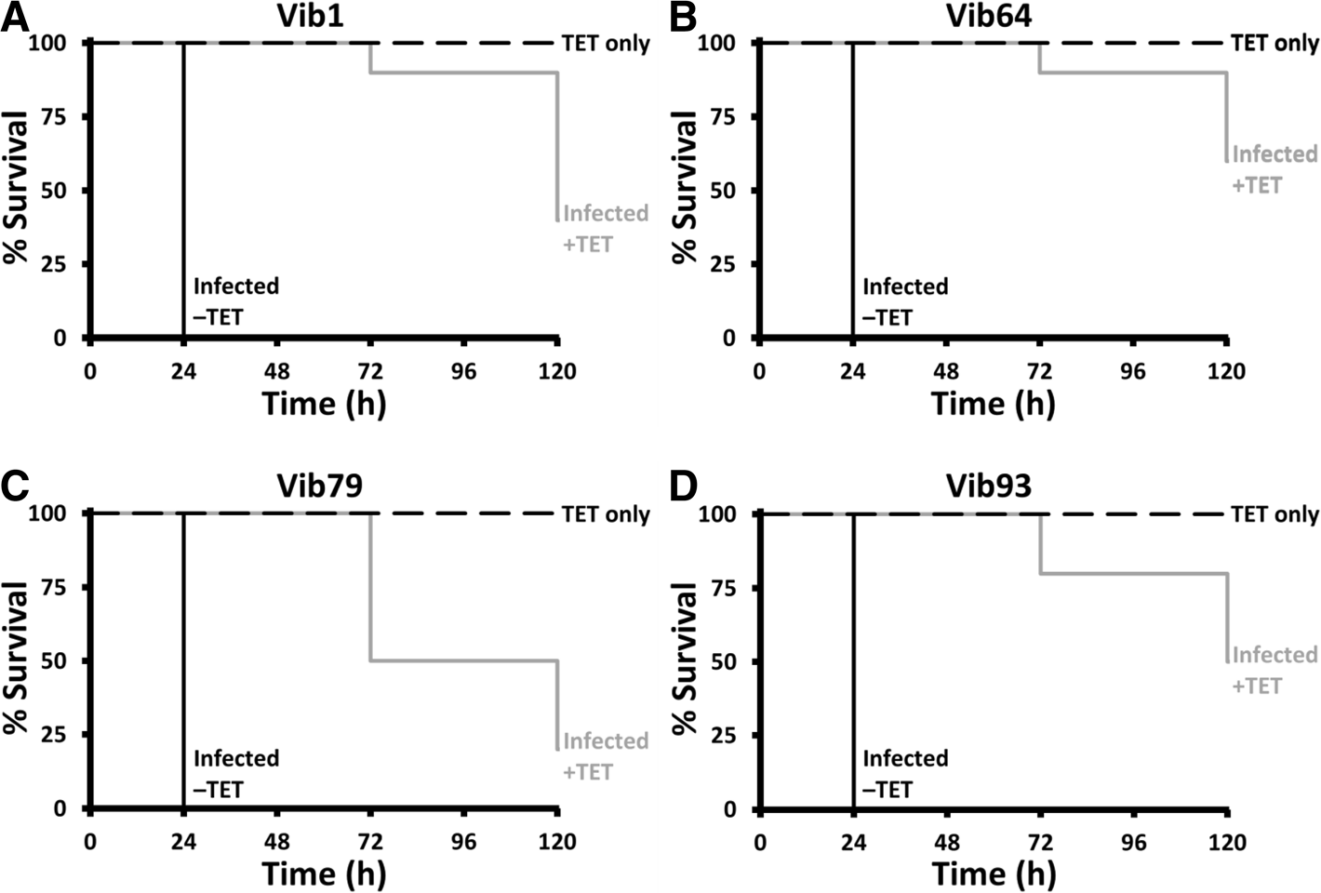
Survival of *Galleria mellonella* larvae injected with 1 × 10^5^ CFU of four representative wild-type *Vibrio anguillarum* isolates and treated at 2 h, 24 h and 48 h with tetracycline (1 μg/g of larva; Infected + TET) in 10 μL phosphate-buffered saline (PBS) during 120 h: Vib1 (**a**), Vib64 (**b**), Vib79 (**c**) and Vib93 (**d**). The infected control group was treated with PBS only (Infected –TET), while the TET only group controlled for the toxicity of the treatments. For clarity, the unmanipulated and uninfected control groups data are not shown. *n* = 10

**Fig. 3:**
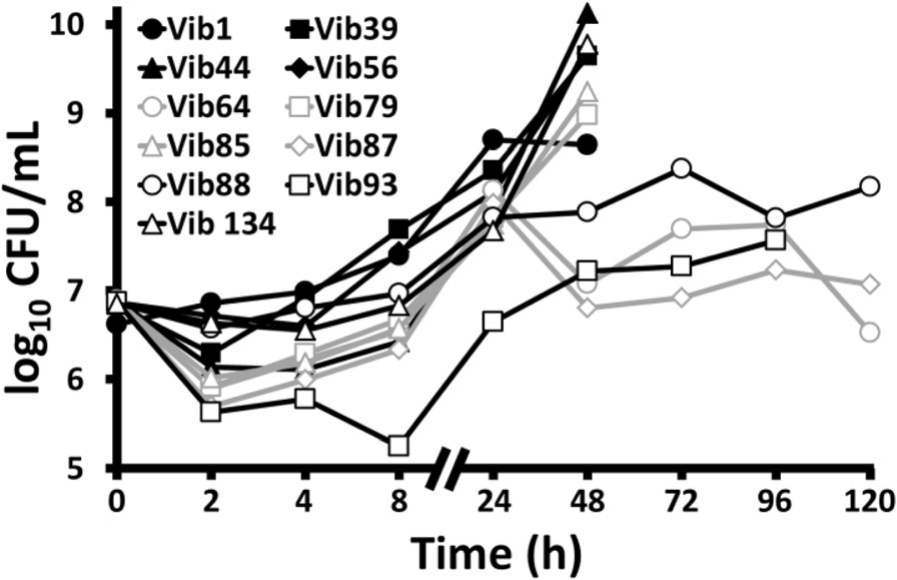
Replication of 11 wild-type *Vibrio anguillarum* isolates in the haemolymph of *Galleria mellonella* larvae during 120 h after injection at 0 h with 1 × 10^5^ CFU. Please note that the CFU/mL value at 0 h refers to the PBS inoculum whereas at the other sampling times the CFU/mL value refers to the haemolymph. It was not possible to obtain data for all strains at each sampling time after 48 h because most larvae were already dead and so were not sampled. For comparative purposes, the geometric mean (and standard error) of CFU/mL in haemolymph for Vib1 at 2 h, 4 h, 8 h, 24 h and 48 h was 6.86 (0.10), 6.99 (0.07), 7.40 (0.17), 8.70 (0.15) and 8.64 (0.85), respectively. For clarity, the unmanipulated and uninfected control groups data are not shown. Data points indicate geometric mean; error bars have not be added. *n* = 4

To assess whether *G. mellonella* would be a suitable model for assessing virulence of *V. anguillarum*, it was necessary to confirm that this bacterium established an infection in the insect. Initial challenge experiments with 11 wild-type isolates demonstrated that there was dose-dependent killing of the larvae for each strain, meaning that greater inocula caused faster and greater mortality in groups (Fig. 1). Injection of larval groups with 5 × 10^7^ CFU heat-killed bacteria had no significant effect on survival during 120 h and typically showed fewer deaths than groups challenged with the lowest dose of live bacteria (1 x10^3^ CFU), suggesting that larval deaths were not occurring due to toxicity of bacterial cells, and viable bacteria were required to cause mortal events (Fig. 1). Moreover, sterile culture filtrate from each isolate had no significant effect on larval survival, indicating that toxic extracellular metabolites were not being produced *in vitro* at sufficiently high concentrations to kill the larvae and viable bacteria were required to bring about death (Fig. 1). For all strains, treatment of infected larvae with 1 μg/g TET gave a significant increase in survival compared to infected larvae treated with PBS only, thus showing that larvae could be rescued from infection with an antibiotic that inhibited the growth of the bacterium (Fig. 2). TET treatment of uninfected larvae had no significant effect on larval survival (Fig. 2). Furthermore, each isolate of *V. anguillarum* showed replication inside the larvae and a group of seven isolates reached ~1 x10^9^ CFU/mL in haemolymph at 48 h while the remaining four isolates largely plateaued at 5 x10^7^ CFU/mL from 24 h (Fig. 3). Taken together, this evidence suggests that viable and replicating *V. anguillarum* cells are needed to establish a systemic infection of *G. mellonella*, and therefore it was next important to see whether virulence of *V. anguillarum* isolates correlated in fish and larva models.

### Positive correlation in virulence of *V. anguillarum* isolates in *S. salar* and *G. mellonella* infection models

**Fig. 4:**
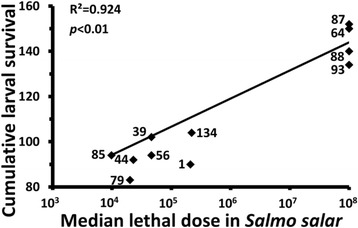
Pearson correlation of virulence of 11 wild-type *Vibrio anguillarum* isolates in the *Galleria mellonella* larvae (calculated as cumulative larval survival; present study) and in a *Salmo salar* infection model (median lethal doses determined after intraperitoneal injection [25]). Median lethal doses for Vib64, Vib87, Vib88 and Vib93 were >10^8^ CFU (the highest dose administered) but these have been plotted as 1 × 10^8^ CFU. The numbers beside the points designate each ‘Vib’ isolate. *n* = 11

The relative virulence of each isolate in the larva was compared with virulence determined previously for each isolate in a *S. salar* infection model [25]. Interestingly, there was highly significant positive correlation (*p* < 0.01) between relative virulence of the 11 wild-type isolates in larva and fish models of infection, indicating that more virulent *V. anguillarum* strains in *S. salar* were also more virulent in the insect (Fig. 4). Closer examination of the growth of each isolate in the larva revealed that more virulent strains replicated faster and reached greater burden in the haemolymph than less virulent isolates (Fig. 3). Indeed, within 48 h the more virulent isolates had increased to approximately 10^9^–10^10^ CFU/mL in the larval haemolymph, whereas less virulent strains reached approximately 10^7^–10^8^ CFU/mL by this time (Fig. 3). After 48 h it was not possible to obtain data for the more virulent strains as most larvae were dead, while the haemolymph burden of less virulent strains remained at approximately 10^7^–10^8^ CFU/mL for the duration of the experiment (Fig. 3). Additionally, mean bacterial burden in the haemolymph of dead larvae was determined and there was always greater than 5.72 × 10^9^ CFU/mL in these insects for the seven strains and times examined, indicating the likely breaching of a burden threshold before larval death ensued (data not shown).

### *V. anguillarum* virulence factors important in fish and *G. mellonella* infections

To assess the role of various *V. anguillarum* virulence factors known to be important in fish infections, the virulence of pairs of parent strains and isogenic knockout mutant strains were compared in the larva model. First, the virulence of *V. anguillarum* NB10 was compared to a strain that had been cured of its pJM1 virulence plasmid, which codes for proteins that scavenge iron and plays a crucial role in virulence of fish infections [32, 33] (Table 2). As expected, the strain cured of its virulence plasmid was significantly less virulent in the larva compared with the parent isolate. In *S. salar*, strains lacking the *rtxA* or *vah1* toxin genes are less virulent than their respective parent isolates, and the reduced virulence of the Δ*rtxA* mutant was reflected in the larva model, though the Δ*vah1* mutant was equally as virulent as its parent (Table 2). A double-knockout strain lacking both *rtxA* and *vah1* genes had attenuated virulence in the larva model, which confirmed earlier observations in fish (Table 2). In fish, inactivation of *empA* (a metalloproteinase possibly important for tissue invasion) reduces virulence, but this same mutant was equally as virulent as its parent strain in the larva (Table 2). Finally, *V. anguillarum* strains lacking functional *flaA*, *flaD*, and *flaE* genes, which are necessary for flagella assembly and aid in adherence and colonisation of fish, show reduced virulence in fish, but these mutants did not show lower virulence in the larva compared with parent strains (Table 2).

## Discussion

Alternative infection models are becoming more important as experimentation on vertebrates becomes increasingly regulated, but few alternative infection models are available to study pathogens of animals produced in aquaculture. Hence, this present study aimed to validate *G. mellonella* as an alternative model to investigate the virulence of *V. anguillarum*, a key aquaculture pathogen that infects many species and reduces farm productivity [21, 22, 24].

In this present study, *V. anguillarum* was shown to establish systemic infections of *G. mellonella* larvae: the bacterium killed the larvae in a dose-dependent manner and replicated *in vivo*, while antibiotics rescued the insect from lethal bacterial inocula. The strains with greater virulence replicated to a greater extent inside the insect haemolymph to bring about faster larval mortality than less virulent isolates, presumably by more effectively combating the innate immune defences to exploit the host. Importantly, there was significant positive correlation between virulence of different wild-type *V. anguillarum* isolates in *S. salar* (native) and *G. mellonella* (alternative) infection models, which is key evidence when validating an alternative host for a particular pathogen, though this is rarely performed or achieved possibly due to the undesirable number of animals required [34, 35]. Pleasingly, the correlation in virulence of *V. anguillarum* isolates in *S. salar* and *G. mellonella* was demonstrated using published virulence data, meaning that no fish were required for this present study [25].

Our data showed that certain virulence determinants were similarly important during fish and insect infections, including the pJM1 virulence plasmid [33] and rtxA (a secreted toxin) [7]; however, some discrepancies were observed for the role of other virulence factors in the models, which is perhaps unsurprising given the physiological differences between the organisms. Indeed, some knockout strains were actually more virulent than parent, for example the Δ*empA* strain. However, Milton et al. [30] first demonstrated a role in virulence for empA in *Oncorhynchus mykiss* infection, but a subsequent study with an Δ*empA* mutant prepared from a different *V. anguillarum* isolate was unable to confirm a role in virulence for this protein in *S. salar* when bacteria were introduced by intraperitoneal injection [36]; a discrepancy that may be due to the use of different host species [36]. The Δ*flaA* and Δ*flaE* flagellum mutants were also more virulent than parent strains in the larva compared with fish. In *V. anguillarum* infections of fish, flagella act in host attachment and dissemination [6, 29] but, given the size of the larva and subversion of requirement for attachment thanks to direct injection into the haemolymph, these organelles may not be required for exploitation of the larva. Moreover, by knocking out flagellum protein genes, metabolic resources might be redirected to other virulence mechanisms that are more important during larva infection. The inability to evaluate the role of attachment and entry virulence factors, which are vital for infection [32], may at first seem to be a limitation of the *G. mellonella* model and indeed it is, however many studies in fish and aquatic invertebrates also rely solely on injected inocula through the external surface to establish infections [7, 25, 37–39]. An additional consideration that might explain the differences observed between fish and larva models is that various fish models were used to confirm the role of a virulence factor and thus comparison to the larva may be considered unfair as we do not know of the relative importance for these factors in different fish species (Table 2). In addition, a recent study of the mammalian pathogen *Candida albicans* in *G. mellonella* also found disparity for the importance of distinct virulence factors in different hosts [40]. These findings do not prevent *G. mellonella* from being useful in virulence studies of *V. anguillarum*, but it does mean that caution is required when extrapolating results or using this model to investigate a specific virulence factor. Therefore, additional work may be needed to identify which particular virulence traits can be studied in this model.

The increasing desire to reduce vertebrate experimentation and adhere to the principles of the 3Rs, while also reducing costs and infrastructure requirements, or other inconveniences associated with studying a pathogen in a native aquaculture host such as strict legislation, mean there are strong incentives for pursuing new more ethically acceptable alternative infection models [3, 4, 14]. Existing alternatives suffer from the need for tissue culture or aquarium facilities, and models may lack immune complexity or be unsuitable for studying certain virulence factors. The *G. mellonella* model is simple to perform, shares functional similarity to many of the innate immune responses of aquaculture species, and permits high-throughput experiments of pathogenicity and virulence factors [14–19]. In this present study, we have validated the *G. mellonella* model for just one aquaculture pathogen (*V. anguillarum*), but this model is likely to be highly suited to the study of virulence in other fish and aquaculture pathogens. Still, the *G. mellonella* model requires full validation for each particular pathogen before its suitability for studying that microbe can be ensured. Crucially, the model does not rely on liquid systems, thus reducing infrastructure requirements and biohazard risks associated with large volumes of contaminated water, which is especially desirable if studying zoonotic pathogens.

## Conclusion

Aquaculture is playing an increasingly important role in global food production and its long-term sustainability relies on the prevention and control of microbial infectious diseases through the development of new and improved treatments, vaccinations and management practices. The use of alternative models such as *G. mellonella* may rapidly improve our understanding of microbial pathogens in aquaculture and lead to the timely introduction of effective remedies for infectious diseases, while considerably reducing the number of vertebrates used in such studies.

### Availability of data and materials

All materials described in this manuscript, including the raw data, are freely available to any scientist wishing to use them for non-commercial purposes and these can be obtained from the Corresponding Author by request.

### Ethics statement

This study was submitted to and approved by the Institute of Aquaculture ethics committee.

## Additional file

Additional file 1: Table S1. Confirmation of mutant strain genotypes: Type of mutation in each strain, primers, expected amplicon length and PCR conditions.
