# Supplementary material for: Larva of the greater wax moth, Galleria mellonella, is a suitable alternative host for studying virulence of fish pathogenic Vibrio anguillarum
Source: BMC Microbiol. 2015 Jun 23;15:127. doi: 10.1186/s12866-015-0466-9 (PMC4477312; doi:10.1186/s12866-015-0466-9)
Supplement: Additional file 1: Table S1. — Confirmation of mutant strain genotypes: Type of mutation in each strain, primers, expected amplicon length and PCR conditions. [file 12866_2015_466_MOESM1_ESM.docx]

**Table S1.** Confirmation of mutant strain genotypes: Type of mutation in each strain, primers, expected amplicon length and PCR conditions.

| Target | Mutation | Sequence of forward primer (5’-3’) | Sequence of reverse primer (5’-3’) | Amplicon length | Annealing temperature (°C) | Reference |
| --- | --- | --- | --- | --- | --- | --- |
| NB12 | *empA*; plasmid insertion | NB12-F: AACAAAAGCAAGCGGTT | pNQ705-R: GCGTAACGGCAAAAGCACCGCCGGACATCA | 638 | 52 | Milton et al. (1992) |
| KD27 | *flaE*; in frame deletion | KD27flaESMF: TATGTATGGTGTGGCGGACG | KD27flaESMR: GCCGCAACATAGCTAAAGCC | 589 | 56 | This study |
| DM16 | *flaA*; in frame deletion | DM16flaASMF: AATCAGCAAACGGCACCAAC | DM16flaASMR: CCTAACCCACGTCTGAGCTG | 735 | 57 | This study |
| KD12 | *flaD*; in frame deletion | KD12flaDSMF: AGCATTGGCCTTACTTCGCT | KD12flaDSMR: TCGCTACACGGTCTGCTTTT | 419 | 57 | This study |
| NB10 cured | Cured of pJM1 virulence plasmid | angRSMF: AAGACGTGACCCGATTGCTT | angRSMR: TATCGATGCTTCGGTGGCTC | 247 | 55 | This study |
| NB10 cured | Cured of pJM1 virulence plasmid | fatESMF: TTTTGTCCATGGCTTCACGC | fatESMR: TGGATGACAAGCACTACGGC | 454 | 57 | This study |
| JR1 | *vah1*; plasmid insertion | vah1JR1SMF: CGTAGTACGGCCAGTATGGT | pNQ705-R: GCGTAACGGCAAAAGCACCGCCGGACATCA | 773 | 63 | F: This study; R: Milton et al. (1992) |
| S123 and S183 | *rtxA*; plasmid insertion | rtxAflanking-F: GAGCCACAGTCTATGGGAAAACCATT | pNQ705.1-R: GCTGTCCCTCCTGTTCAGCTACTGAC | 559 | 63 | Prof. D. Nelson, Pers. Comm. |
| S183 | *vah1*; in frame deletion | vah1S183SMF: ATAATTCGCCACAAAGGTGCC | vah1S183SMR: CTTACTTAACGCCACCGTGC | 2534 | 56 | This study |

**Milton DL, Norqvist A, Wolf-Watz H.** 1992. Cloning of a metalloprotease gene involved in the virulence mechanism of *Vibrio anguillarum*. J. Bacteriol. **174:**7235–7244.
